# Associations between Thyroid Hormones, Calcification Inhibitor Levels and Vascular Calcification in End-Stage Renal Disease

**DOI:** 10.1371/journal.pone.0132353

**Published:** 2015-07-06

**Authors:** Christiaan Lucas Meuwese, Hannes Olauson, Abdul Rashid Qureshi, Jonaz Ripsweden, Peter Barany, Cees Vermeer, Nadja Drummen, Peter Stenvinkel

**Affiliations:** 1 Department of Renal Medicine, CLINTEC, Karolinska Institutet, Stockholm, Sweden; 2 Medical Imaging and Technology, CLINTEC, Karolinska Institutet, Stockholm, Sweden; 3 VitaK, Maastricht University, Maastricht, The Netherlands; Brigham and Women's Hospital, Harvard Medical School, UNITED STATES

## Abstract

**Introduction:**

Vascular calcification is a common, serious and elusive complication of end-stage renal disease (ESRD). As a pro-calcifying risk factor, non-thyroidal illness may promote vascular calcification through a systemic lowering of vascular calcification inhibitors such as matrix-gla protein (MGP) and Klotho.

**Methods and Material:**

In 97 ESRD patients eligible for living donor kidney transplantation, blood levels of thyroid hormones (fT3, fT4 and TSH), total uncarboxylated MGP (t-ucMGP), desphospho-uncarboxylated MGP (dp-ucMGP), descarboxyprothrombin (PIVKA-II), and soluble Klotho (sKlotho) were measured. The degree of coronary calcification and arterial stiffness were assessed by means of cardiac CT-scans and applanation tonometry, respectively.

**Results:**

fT3 levels were inversely associated with coronary artery calcification (CAC) scores and measures of arterial stiffness, and positively with dp-ucMGP and sKlotho concentrations. Subfractions of MGP, PIVKA-II and sKlotho did not associate with CAC scores and arterial stiffness. fT4 and TSH levels were both inversely associated with CAC scores, but not with arterial stiffness.

**Discussion:**

The positive associations between fT3 and dp-ucMGP and sKlotho suggest that synthesis of MGP and Klotho is influenced by thyroid hormones, and supports a link between non-thyroidal illness and alterations in calcification inhibitor levels. However, the absence of an association between serum calcification inhibitor levels and coronary calcification/arterial stiffness and the fact that MGP and Klotho undergo post-translational modifications underscore the complexity of this association. Further studies, measuring total levels of MGP and membrane bound Klotho, should examine this proposed pathway in further detail.

## Introduction

Patients with chronic kidney disease (CKD) are exposed to a greatly increased risk of cardiovascular morbidity and mortality compared to the general population[1] Underlying mechanisms linking CKD to CVD are incompletely understood but encompass both traditional and novel risk factors.[1, 2] In contrast to the situation in the general population, the predominant vascular pathology in CKD is arterial media calcification.[3]

In the genesis of uremic vascular calcification, osteochondrocytic differentiation of vascular smooth muscle cells (VSMC) has appeared as a cornerstone process.[4] The osteoblast like VSCMCs produce bone proteins and release pre-calcified membrane matrix vesicles,[4] which normally contain calcification inhibitors, such as Matrix Gla protein (MGP), preventing them to exert their calcifying actions.[5] In order to act as a calcification inhibitor, MGP must first be activated by posttranslational gammaglutamate carboxylation. As this process is vitamin K dependent and patients with ESRD typically have a poor vitamin K status, plasma levels of desphospho-uncarboxylated MGP (dp-ucMGP) are generally elevated.[6] In more severe states of vitamin K insufficiency, also blood-clotting factors are affected and uncarboxylated clotting factors, or PIVKAs (Proteins Induced by Vitamin K Absence), are detectable in the circulation. The most commonly detected PIVKA is descarboxyprothrombin, also known as PIVKA-II.

Another recently discovered factor of interest is Klotho, a membrane-bound protein expressed at the highest levels in renal tubules, parathyroid glands and choroid plexus. Membrane-bound Klotho functions as co-receptor for Fibroblast growth factor-23 (FGF23), allowing for high-affinity binding to FGF-receptors.[7] FGF23-Klotho signalling is essential for phosphate and vitamin D homeostasis, and is severely dysregulated in CKD. Klotho can also be shedded from the cell surface into circulation by α-secretases to form soluble Klotho (sKlotho). In vitro, sKlotho was demonstrated to inhibit sodium-dependent phosphate uptake in VSMC and thereby prevent phosphate-induced vascular calcification.[8]

Finally, hormonal derangements in ESRD include a systemic lowering of serum free triiodothyronine (fT3) and thyroxine (fT4) concentrations, making up part of the non-thyroidal illness spectrum.[9] Presence of non-thyroidal illness in ESRD has been strongly associated with cardiovascular mortality,[10] and also with vascular calcification,[11–13] whereby posing it as a candidate cardiovascular risk factor that can be manipulated in ESRD. These observations are reinforced by studies in the general population showing associations between subclinical thyroid hormone alterations and an increased coronary calcification.[14] The increased cardiovascular risk due to non-thyroidal illness could be explained by the promotion of endothelial dysfunction, vasoconstriction and lipid alterations by a systemic low thyroid hormone state.[15] These pathways seem, however, not fully able to explain the specific presence of media calcification.

**Fig 1:**
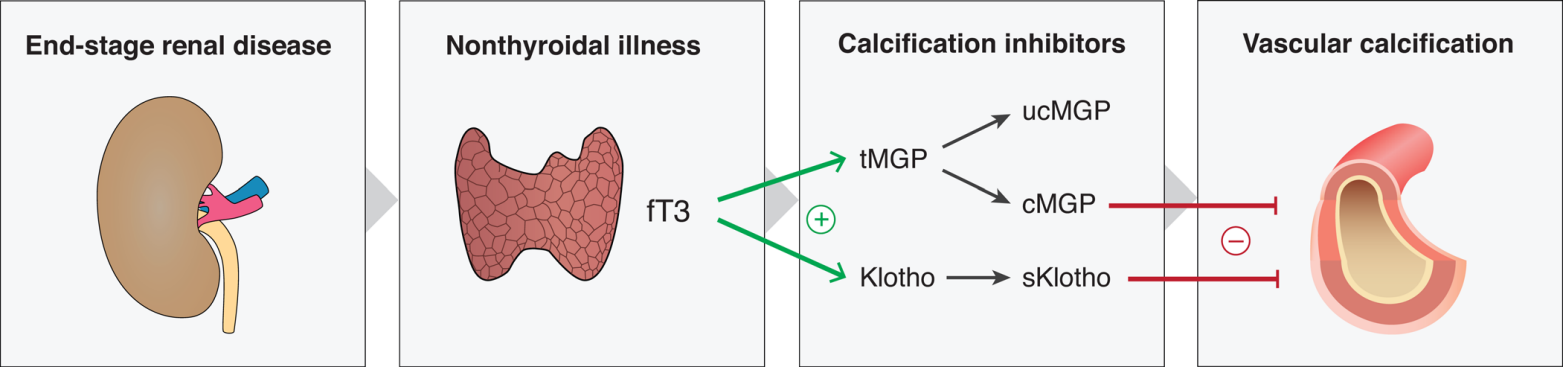
A hypothesis on the impact of non-thyroidal illness on vascular calcification.

Recent in vitro studies have suggested functional links between thyroid hormones, MGP and Klotho. First, Sato et al.[16] observed that physiological concentrations of T3 facilitate MGP gene expression in smooth muscle cells, an effect that is likely mediated by thyroid hormone response element in the promotor region of the MGP gene.[16] Similarly, Klotho synthesis was reported to be under control of thyroid hormone stimulation.[17] These findings lead us to speculate that nonthyroidal illness could initiate an increased vascular calcification through down-regulation of the protective effects by MGP and Klotho (Fig 1). Therefore, we studied; 1) the association between thyroid hormone concentrations and (i) vascular calcification/arterial stiffness, (ii) sKlotho and (iii), MGP as well as; 2) the association of plasma MGP and sKlotho concentrations with surrogate markers of vascular calcification/arterial stiffness in a carefully phenotyped cohort of younger ESRD patients.

## Methods and Materials

### Patients

Between 2009 and August 2014 adult ESRD patients selected to undergo living donor kidney transplantation (LD-RTx) at Karolinska University Hospital in Stockholm, Sweden, were invited to participate in the study. Ethical approval for the study protocol was obtained from the regional ethical committee in Stockholm County, Sweden who approved the consent procedure. Patients provided written informed consent. Out of 101 patients who met the inclusion criteria and gave informed consent, two were excluded because of warfarin usage and another two due to incomplete thyroid hormone data, leaving 97 patients for the present study. Of these 97 patients, the most common causes of CKD were polycystic kidney disease (n = 18) glomerulonephritis (n = 29), diabetic nephropathy (n = 6), interstitial nephritis (n = 7), other causes (n = 23), and unknown (n = 14). The most commonly used forms of medication were erythropoietin (78.4%), beta-blockers (55.7%), non-calcium-based phosphate binders (53.6%) and loop-diuretics (60.9%). CVD was defined as having coronary artery, cerebrovascular, and/or peripheral artery disease. Subjective global assessment (SGA) was determined as previously exemplified.[18]

### Laboratory measurements

Venous blood was collected after an overnight fast; all blood samples were centrifuged immediately and stored at -70°C until required for analysis. Serum high-sensitivity C-reactive protein (CRP), calcium, phosphate, creatinine, and albumin levels were determined using routine methods at the Department of Laboratory Medicine, Karolinska University Hospital. Serum levels of IL-6 were quantified using an Immulite system (Siemens Healthcare Diagnostics, Los Angeles, CA, USA). Plasma analyses of thyroid hormones were performed using a Roche Modular E/Cobas E analyser and commercially available electrochemiluminescence immunoassays for fT3 [analytical sensitivity (AS), 0.4 pmol/L; total coefficients of variation (CVs), 6.27% and 3.36% at 3.16 and 10.03 pmol/L, respectively], fT4 (AS, 0.3 pmol/L; total CVs, 2.05% and 3.03% at 12.01 and 34.70 pmol/L, respectively) and TSH (AS, 0.014 mU/L; total CVs, 2.16% and 2.42% at 0.995 and 5.530 mU/L, respectively). Results are expressed as the average of two measurements.

Vitamin K status was assessed by measuring dp-ucMGP levels in citrate plasma using the InaKtif MGP iSYS kit (IDS, Boldon), a pre-commercial automated assay based on the conformation-specific dual antibody ELISA. [6] Total uncarboxylated MGP (t-ucMGP) was measured using a competitive (single antibody) ELISA, in which the capture antibody is directed against ucMGP, and plasma ucMGP is quantified from its competition with a labelled tracer peptide.^7^ Both assays were performed at VitaK, Maastricht University, The Netherlands. Plasma concentrations of PIVKA-II were determined by an enzyme immunoassay (Eisai Co., Tokyo, Japan) according to the manufacturer’s instructions. Soluble Klotho levels were determined using a commercially available ELISA kit (Immuno-Biological Laboratories Co., Ltd, Japan) according to the manufacturer’s instructions.

### Cardiac CT-scans

Cardiac CT scans were performed using a 64-channel detector scanner [LightSpeed VCT; General Electric (GE) Healthcare, Milwaukee, WI, USA] in cine mode. Scans were ECG-gated and a standard non-contrast protocol was used with a tube voltage of 100 kV, tube current of 200 mA, 350 ms rotation time, 2.5 mm slice thickness and displayed field of 25 cm. Data were processed and analysed using an Advantage Workstation 4.4 (GE Healthcare). Smartscore 4.0 (GE Healthcare) was used to assess CAC scores. Calcified plaques were considered to be present if values crossed the standard threshold of 130 Hounsfield units. CAC scores were expressed in Agatston units (AU) as previously described in detail.[19] Total CAC score was calculated as the sum of the CAC scores in the left main artery, the left anterior descending artery, the left circumflex artery and the right coronary artery.

### Arterial Stiffness

Arterial elasticity was assessed by means of applanation tonometry (SphygmoCor device, AtCor Medical, Sydney, Australia). Measurements were conducted in a climate-controlled room and after patients had rested for five minutes in a supine position. On basis of 20 consecutive measurements at the radial site, the device constructs a peripheral arterial waveform, which is transcalculated into an aortic waveform using a validated transfer formula [20]. Aortic systolic and diastolic pressures and pulse pressure were calculated on basis of these derived aortic wave shapes. The difference between the first and second systolic peaks of the aortic waveform defined the aortic augmentation pressure. The ratio of diastolic and systolic pressure time index defined Buckberg’s subendocardial viability ratio (SEVR).

### Statistical analyses

Patient characteristics were presented across groupings based on tertiles of fT3 (cut-off values: 2.73 and 3.71 pmol/L) using means and standard deviations (SD), medians and interquartile ranges (IQR) and numbers and percentages, accordingly. Linear trends across groups were tested by means of the appropriate tests.

Associations between thyroid hormone levels and arterial stiffness and calcification inhibitor parameters were examined by means of scatter plots and linear regression analyses. Because of non-normality, t-ucMGP, dp-ucMGP, PIVKA, and sKlotho levels were logarithmically transformed. Models were adjusted for age, sex, diabetes mellitus, dialysis vintage, SGA, and IL-6 levels. The association between fT3 levels and CAC scores was studied using logistic regression analyses. For this purpose, CAC scores were dichotomized across 400 AU. In these analyses, age sex, and diabetes mellitus were considered as potential confounders. The cut-off values of 100 and 400 AU were chosen on basis of the distribution of our population and previous recommendations.[21] For illustrational purposes, histograms were created showing the percentage of patients having CAC scores over 100 and 400 AU across tertiles of fT3 distribution. The coherence between the different systemic calcification inhibitors and thyroid hormones was studied using Spearson correlation coefficients.

Statistical analyses were performed using STATA version 12.1 (StataCorp LP, Texas, USA). Betas with 95% confidence intervals (CIs) not containing 0, and odds ratios with 95% CIs not including 1 were considered statistically significant. For all other tests, a p-value lower than 0.05 was considered to indicate statistical significance. Figures were created using PRISM 5.02 (GraphPad Software Inc, La Jolla, CA, USA 1992).

## Results

### Baseline and outcome characteristics

The cohort comprised 97 individuals with an average (SD) age of 45.1 (14.0) years. Sixty three (64.9%) of patients received dialysis treatment for a median (IQR) duration of 1.0 (0.5–2.0) years before LD-Rtx. The others underwent preemptive Rtx. Patients in the lowest fT3 tertile had more frequently pre-existing CVD and on average lower albumin and phosphate levels as compared with the higher tertiles (Table 1). Calcium levels were not significantly different between groups.

**Table 1:** Baseline characteristics according to fT3 tertiles.

	Lowest tertile		Middle tertile	Highest tertile	p-for trend
	fT3 < 2.73 pmol/L		2.73 > fT3 < 3.71 pmol/L	fT3 > 3.71 pmol/L	
	n = 32		n = 32	n = 33	
**General characteristics**					
** Men, n %** ^**¶**^	17 (53.1)		19 (59.4)	26 (78.8)	0.095
** Age, years** ^**#**^	46.2 (13.9)		44.5 (14.7)	44.5 (13.8)	0.414
** CVD, n %** ^**¶**^	9 (28.1)		4 (12.5)	5 (15.2)	0.128
** Diabetes Mellitus, n %** ^**¶**^	10 (31.3)		4 (12.5)	5 (15.2)	0.067
** SGA, n %** ^**1**^ ^**,**^ ^**¶**^	9 (30)		9 (29)	3 (9.7)	0.121
** Previously on dialysis, %**	19 (59.4)		22 (67.8)	22 (66.7)	0.789
** Dialysis vintage, years** ^**2**^ ^**,**^ ^*^	1.0 (0.4–2.0)		0.7 (0.5–1.3)	1.0 (0.5–2.7)	0.709
**Medication**					
** Beta-blockers, n %** ^**¶**^	17 (53.1)		20 (62.5)	17 (51.5)	0.987
** Phosphate binders, n %** ^**¶**^	18 (56.3)		17 (53.0)	17 (51.5)	0.810
**Laboratory measurements**					
** Albumin, g/L** ^**#**^	34.7 (3.0)		36.0 (3.6)	37.3 (3.5)	0.005
** Interleukin-6, pg/L**	1.1 (0.5–1.8)		0.9 (0.4–2.2)	1.3 (0.8–2.1)	0.429
** PTH, pg/mL**	296 (218–439)		222 (96–360)	196 (156–449)	0.118
** Calcium, mmol/L** ^**#**^	2.3 (0.2)		2.3 (0.2)	2.3 (0.3)	0.541
** Phosphate, mmol/L** ^**#**^	1.5 (0.3)		1.8 (0.7)	1.7 (0.5)	0.031
**Thyroid hormone profile**					
** TSH, mIU/L** ^*^	0.75 (0.30–1.05)		0.76 (0.42–0.96)	1.20 (0.63–1.67)	0.106
** fT3, pmol/L** ^**#**^	2.24 (0.37)		3.22 (0.27)	4.30 (0.53)	-
** fT4, pmol/L** ^**#**^	15.27 (4.68)		15.39 (2.33)	14.92 (2.13)	0.801

1. SGA: subjective global assessment > 1.

2. Vintage was calculated in those who were previously on dialysis.

* Medians plus interquartile ranges (IQR). Differences were tested by means of a non-parametric trend test.

# Means plus standard deviations (SD). Differences were tested by means of univariate regression analyses

¶ Numbers and percentages. Differences were tested by means of Chi squire tests.

### Thyroid hormones and arterial stiffness/vascular calcification

In Table 2, the distribution of arterial stiffness parameters across fT3 tertiles is presented. With decreasing fT3 levels, patients had significantly higher pulse pressure (PP) and tended to have lower aortic augmentation pressure (AP). Regression analyses in Table 3 show a significant inverse association between fT3 levels and PP (β [95%CI]: -4.2 [-7.3 to -1.1] p = 0.008), which sustained after adjustment for confounding factors (-3.5 [-6.7 to -0.3] p = 0.031). For the association between fT3 and diastolic BP and Aortic AP, a trend was seen (-1.4 [-2.9 to 0.0] p = 0.052) while no such link became apparent for the associations between fT3 and systolic BP, Aortic augmentation index (Aix) and SEVR.

**Table 2:** Vascular calcification characteristics of patients according to fT3 status.

	Lowest tertile	Middle tertile	Highest tertile	p-for trend
	fT3 < 2.67 pmol/L	fT3 2.67–3.79 pmol/L	fT3 > 3.79 pmol/L	
	n = 32	n = 32	n = 33	
**Arterial stiffness**				
** Systolic BP, mmHg** ^**#**^	142 (24)	138(18)	140(20)	0.531
** Diastolic BP, mmHg** ^**#**^	80 (12)	82(11)	86(11)	0.058
** Pulse pressure, mmHg** ^**#**^	63(16)	57(13)	54(14)	0.014
** Aortic AP, mmHg** ^*^ ^**,**^ ^**1**^	7.0(4.0–12.0)	7.0(3.0–10.0)	6.0(0.0–11.0)	0.079
** Aortic Aix, mmHg** ^*^ ^**,**^ ^**1**^	19.0(12.0–23.0)	20.0 (9.0–25.0)	16.0(1.0–25.0)	0.388
** SEVR, %** ^*^ ^**,**^ ^**1**^	149.2(29.7)	137.0(28.6)	150.8(33.8)	0.830
**Serum calcification inhibitors**				
** t-ucMGP, nmol.L** ^*^ ^**,**^ ^**1**^	2932(2017–3737)	2690(2160–3507)	2703(1800–3553)	0.675
** dp-ucMGP, pmol/L** ^*^ ^**,**^ ^**1**^	1543(1194–2082)	1723(1274–2419)	2301(2011–3428)	0.001
** PIVKA-II, mAU/mL** ^*^ ^**,**^ ^**2**^	39.5(26.0–54.0)	32.0(21.0–44.0)	43.0(28.5–56.0)	0.675
** sKlotho, pg/ml** ^*^	315(239–513)	324(225–414)	421(363–561)	0.129

BP; Blood pressure, AP: aortic augmentation pressure, Aix: aortic augmentation index, SEVR: Subendocardial viability ratio.

1. Measurements were available in 28, 24 and 27 patients respectively.

2. Measurements were available in 18, 19 and 24 patients respectively.

* Medians plus interquartile ranges (IQR). Differences were tested by means of a non-parametric trend test.

# Means plus standard deviations (SD). Differences were tested by means of univariate regression analyses

**Table 3:** Regression analyses on the associations between fT3 and measures of arterial stiffness and calcification inhibitors.

	Crude model		Adjusted^1^	
	Beta (95%CI)	p-value	Beta (95%CI)	p-value
**Linear regression analyses**				
**Arterial stiffness**				
** Systolic BP** ^**#**^	-1.8(-6.2 to 2.6)	0.423	-1.6(-6.2 to 3.0)	0.485
** Diastolic BP**	2.4(-0.2 to 5.0)	0.066	1.9(-0.3 to 9.5)	0.067
** Pulse pressure**	-4.2(-7.3 to -1.1)	0.008	-3.5(-6.7 to -0.3)	0.031
** Aortic AP**	-1.4(-2.9 to 0.0)	0.052	-1.3(-2.8 to 0.2)	0.081
** Aortic Aix**	-1.8(-4.2 to 0.7)	0.153	-1.8(-4.3 to 0.7)	0.159
** SEVR**	1.8(-5.4 to 9.0)	0.624	0.8(-7.6 to 9.1)	0.858
**Laboratory parameters**				
** Log(t-uc-MGP)**	-0.04(-0.16 to 0.07)	0.443	0.00(-0.13 to 0.13)	0.971
** Log(dp-uc-MGP)**	0.15(0.05 to 0.25)	0.004	0.13(0.02 to 0.24)	0.019
** Log(PIVKA)**	0.06(-0.06 to 0.19)	0.312	0.05(-0.10 to 0.21)	0.482
** Log(sKlotho)**	0.12(0.01 to 0.22)	0.031	0.09(-0.04 to 0.21)	0.187
**Logistic regression analyses** ^**2**^				
** CAC > 100**	0.58(0.31 to 1.09)	0.090	0.34(0.13 to 0.92)	0.033
** CAC > 400**	0.35(0.14 to 0.88)	0.026	0.19(0.05 to 0.74)	0.017

BP; Blood pressure, AP: aortic augmentation pressure, Aix: aortic augmentation index, SEVR: subendocardial viability ratio.

1 Adjusted for sex, age, diabetes mellitus, IL-6, vintage, and SGA.

2 The logistic regression analyses for the association between fT3 and CAC scores were adjusted for age and sex.

**Fig 2:**
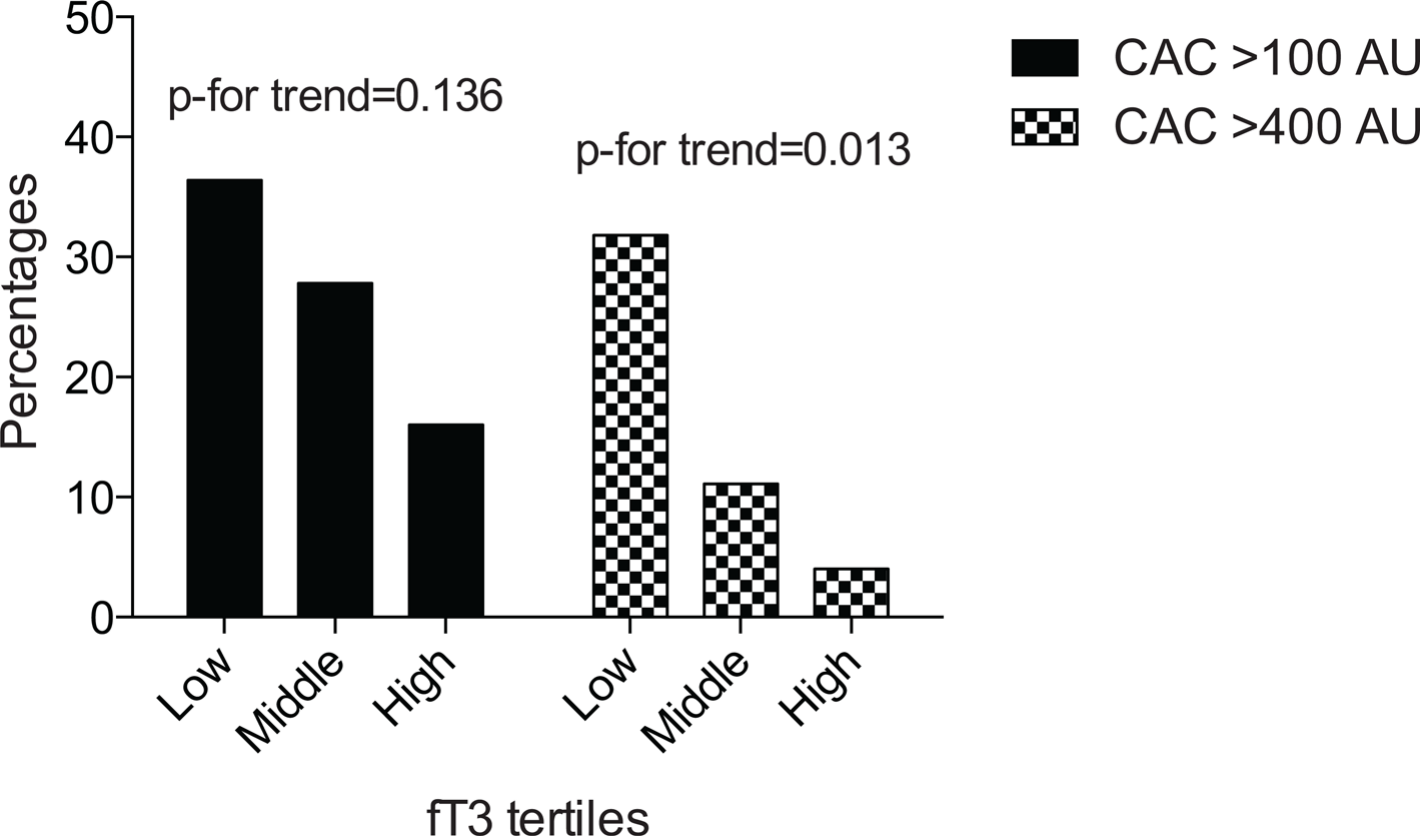
Coronary artery calcification scores across tertiles of fT3.

Cardiac CT-scans (n = 65) showed that 26.2, 15.4 and 13.9 percent of patients had CAC scores over 100, 400 and 800 AU, respectively. A dose response association was observed between fT3 tertiles and CAC scores; a significantly larger part of patients in the lower fT3 tertiles vs. those in the highest tertile had CAC scores >400 AU (Fig 2). Logistic regression analyses confirmed this association and showed that, per pmol/L increase in fT3, the adjusted odds [95% CI] for having CAC scores >400 AU was 0.19 [0.05–0.74, p = 0.017] lower. Linear regression analyses showed an average (95% CI) decrease in CAC scores (β: -179 [: -356 to -1] p = 0.048) with an increment of one pmol/L in fT3 concentration, persisting after adjustment (β: -164 [-329 to 1] p = 0.051). TSH levels showed an inverse association with CAC scores; (per logTSH increase, adjusted odds for having CAC levels >100 AU and >400 AU decreased with 0.3 [0.1–0.8, p = 0.019] and 0.4 [0.1–1.0, p = 0.049], respectively (S1 Appendix)). Higher fT4 levels associated with an increased adjusted odds for having CAC scores > 100 AU by 13 [1.0–1.6, p = 0.021]. Neither TSH nor fT4 levels were associated with parameters of vascular stiffness (S1 Appendix).

### Thyroid hormones and plasma calcification inhibitors

**Fig 3:**
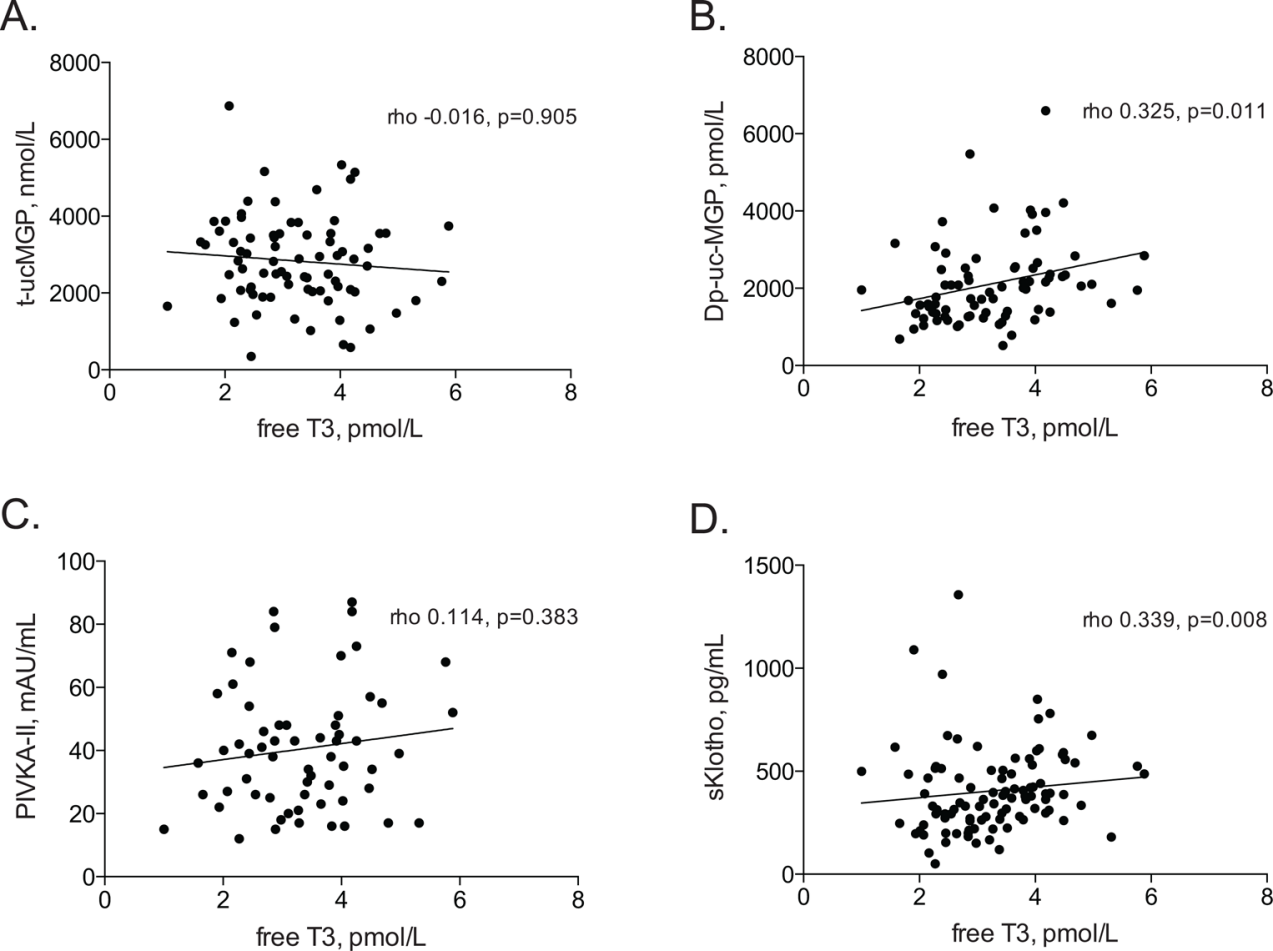
Associations between fT3 and t-ucMGP (A), dp-ucMGP (B), PIVKA (C), and sKlotho (D).

With an increase in fT3 tertile, and although not statistically significant, a gradual increase in plasma levels of dp-uc-MGP and sKlotho was noted (Table 2). Plasma levels of t-ucMGP, and PIVKA did not differ between the different fT3 tertiles. Fig 3 illustrates scatterplots of the associations between fT3 and t-ucMGP (A), dp-ucMGP (B), PIVKA-II (C) and sKlotho (D). As shown in Table 3, results from linear regression analyses confirmed a statistically significant association between fT3 and dp-uc-MGP (β: 0.15 [0.05 to 0.25] p = 0.004) and between fT3 and sKlotho (β: 0.12 [0.01 to 0.22] p = 0.031). Only the association between fT3 and Log(dp-ucMGP) persisted after adjustment for age, sex, diabetes mellitus, cardiovascular disease, vintage, IL-6 levels, SGA, PIVKA-II and albumin levels. FT3 levels were not significantly associated with t-uc-MGP and PIVKA-II. fT4 and TSH concentrations were not associated with calcification inhibitor levels (S1 Appendix). As expected, PIVKA-II associated with dp-ucMGP (Rho = 0.32, p = 0.01).

### Plasma calcification inhibitors and arterial stiffness/vascular calcification

**Fig 4:**
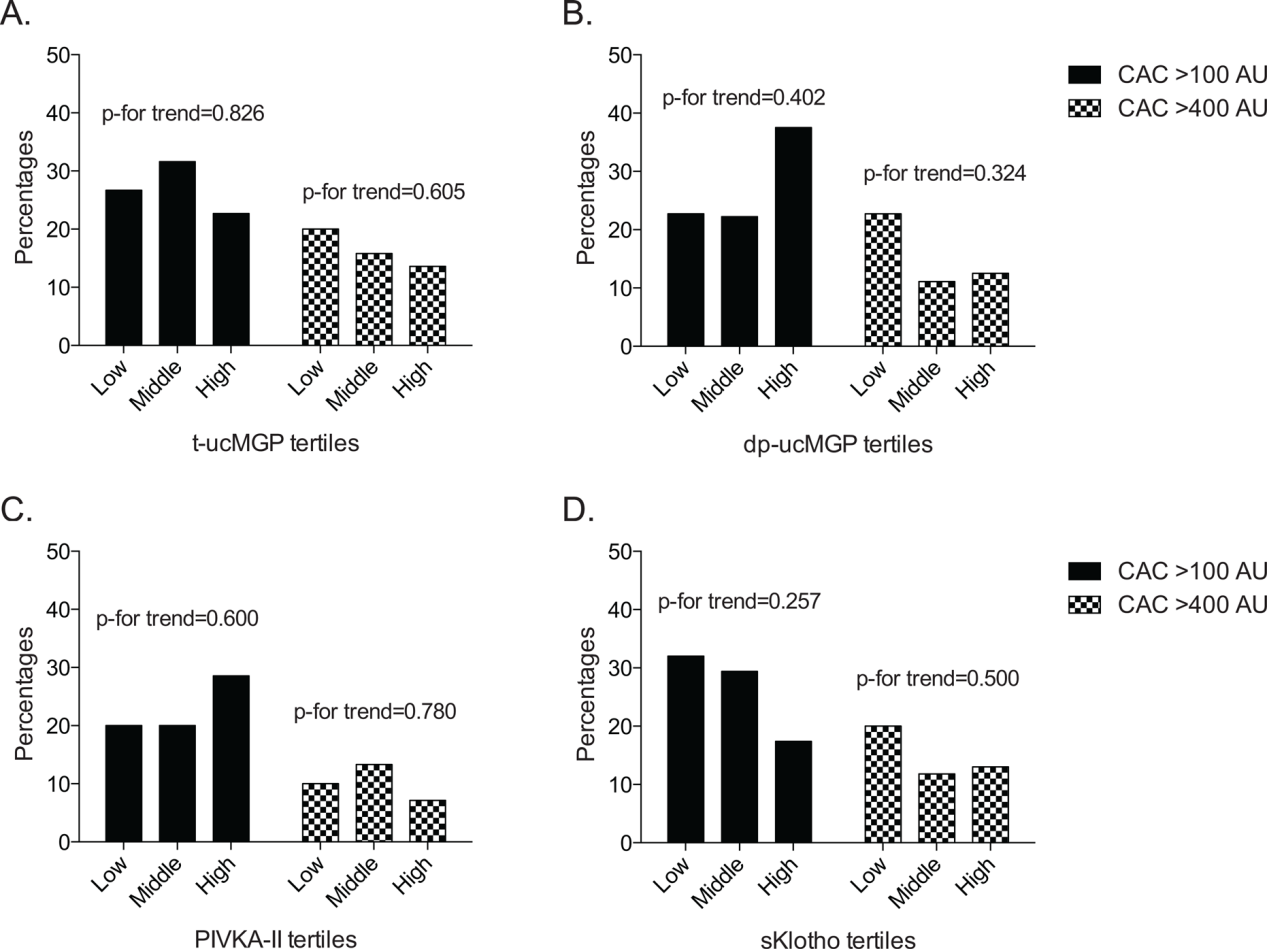
Percentage of patients with elevated CAC scores > 100 and > 400 across t-ucMGP (A), dp-ucMGP (B), PIVKA (C), and sKlotho (D) tertiles.

Fig 4 illustrates percentages of individuals with CAC scores over 100 and 400 AU across tertiles of t-ucMGP (A), dp-ucMGP (B), PIVKA-II (C), and sKlotho (D). For sKlotho, a larger part of patients in the lower tertiles had CAC scores >400 AU, although this association did not reach statistical significance (p-for trend = 0.226). With regards to measures of arterial stiffness we found that higher dp-ucMGP levels were associated with a higher aortic AP in crude (β: 3.7 [0.2 to 6.8] p = 0.021) but not in adjusted models (β: 2.2 [-1.0 to 5.4] p = 0.179). No other associations between plasma calcification inhibitors and vascular calcification/arterial stiffness were found.

## Discussion

In a selected group of younger ESRD patients, fT3 levels were inversely associated with CAC scores and measures of arterial stiffness. Moreover, fT3 levels were positively associated with circulating levels of dp-uc-MGP and Klotho. However, neither sKlotho, dp-ucMGP nor PIVKA-II were associated with CAC scores and arterial stiffness.

We also report markedly elevated median PIVKA-II and dp-ucMGP levels and that these markers for vitamin K status (hepatic and vascular vitamin K status, respectively) were positively associated.[22] Thus, our data support previous literature that ESRD patients have severe vitamin K deficiency and are at high risk of vascular calcification.[23] Average circulating levels of dp-ucMGP (measured in the same laboratory) were reported to be markedly lower (about 400 pmol/L) in healthy volunteers of the same age group. [24] Impaired carboxylation of MGP (i.e. high levels of dp-ucMGP) has previously been reported to be associated with intimal and medial vascular calcification also in other patient populations prone to calcification, such as patients with aortic stenosis [25]. Although circulating levels of t-ucMGP were markedly lower in ESRD patients investigated in the present study than in healthy controls [6], we report no difference in CAC scores and grades of arterial stiffness across the range of t-ucMGP levels.

The proportion of patients with elevated CAC scores was lower in the current cohort than in previous studies in patients on peritoneal dialysis (PD) [13, 26, 27] and hemodialysis (HD) [28]. This divergence could be explained by the selective nature of our cohort as LD-RTx recipients are selected by virtue of their younger age, relatively good physical condition and low comorbidity burden. Also, they have often spent little or no time on maintenance dialysis. A comparison of baseline characteristics between the different cohorts indeed supports this reasoning. Our data show that fT3 levels were inversely associated with CAC scores and arterial stiffness, a finding consistent with our previous report in prevalent dialysis patients.[13] In adjusted analyses, we also observed a statistically significant association in which lower TSH levels were accompanied by higher CAC scores. This observation fits the concept of non-thyroidal illness in which a general down-regulation of the hypothalamic-pituitaric-thyroidal (HPT)-axis occurs in more severe forms and a fT3 levels corresponds to lower TSH levels.[29]

Given prior observations of an up-regulation of MGP [16] and Klotho [17] by stimulation of fT3 in vitro and their established function as calcification inhibitors, we hypothesized on a possible intermediating role of MGP and sKlotho in the causal path between non-thyroidal illness and vascular calcification (Fig 1). The current study is the first to address this hypothesis in the setting of uremia. We observed that fT3 levels were positively associated with dp-ucMGP, supporting the hypothesis of an increased expression of MGP upon fT3 stimulation as suggested by Sato et al. [16]. Combined with limited availability of vitamin K in the uremic milieu the extra synthesis of MGP, driven by fT3, could lead to higher levels of dp-ucMGP. Intriguing in the interpretation of this association is that the uremic milieu is typified by elevated dp-ucMGP rather than low levels [6], which in turn have been associated with higher mortality hazards.[30] This association could therefore not intermediate the link between non-thyroidal illness and an increased vascular calcification in ESRD and implies much more complex interactions. Intervention studies with thyroid hormones in vitamin K-replete controls and vitamin K deficient uremic subjects to study changes in total and dp-ucMGP may be one way to resolve this.

In this selected cohort, t-ucMGP and dp-ucMGP levels were not associated with surrogate markers of vascular calcification, such as CAC score, or arterial stiffening, which contrasts to previous findings in older and sicker ESRD patients.[31, 32] When directly scoring degree of medial calcification in arterial biopsies however, significantly higher levels of uc-dpMGP were found in younger ESRD patients with moderate and severe vascular calcification assessed by histology of abdominal arterial biopsies (unpublished observation Stenvinkel et al. 2015). It could be speculated that CAC score reflects intimal coronary calcification whereas dp-ucMGP concentrations are more associated with the risk of medial vascular calcification. Published studies demonstrate a high sensitivity of CAC for the presence of coronary artery disease but a lower specificity for obstructive CAC. [33] Moreover, previous autopsy studies have shown that the correlation between femoral and coronary atherosclerosis was weak, especially in younger individuals. [34, 35] Finally, although higher dp-ucMGP predicted higher total, non-cancer and cardiovascular mortality in a recent study of 2318 participants from a Flemish population study, it predicted a lower risk for coronary events [36]. Although this discrepant finding may reflect bias due to competing events, it may also imply a differential impact on different outcomes.

In the present study fT3 levels were significantly associated with sKlotho concentrations, a finding that partly accords with a previous in vitro study on murine adipocytes showing an increased synthesis of the membrane-bound form of Klotho upon T3 stimulation.[17] The same study however failed to observe a concomitant increase in sKlotho levels.[17] We have previously demonstrated that the kidney is the principal source of sKlotho, and that renal expression of membrane-bound Klotho correlate closely to sKlotho levels.[37] However, in the uremic milieu the correlation between the membrane-bound isoform and sKlotho is weak or absent and most studies have failed to find a consistent correlation between sKlotho and renal function.[38] This could speculatively be explained by altered shedding of membrane-bound Klotho in the uremic milieu, or by methodological problems with the current assays. In our analyses, a weak inverse trend was observed between sKlotho tertiles and CAC scores, which accords with an animal study describing higher vascular calcium content in Klotho-haploinsufficient mice with CKD versus wild type equivalents.[8] In contrast, sKlotho levels were not independently associated with the presence of cardiovascular disease in a Dutch sample of 127 dialysis patients.[39] Nevertheless, the current association between thyroid hormones and the calcification process implicates a role for fT3 in regulation of sKlotho. In line with this, a recent study in rats with calcified aortas due to the effects of nicotine and vitamin D3 found that T3 supplementation resulted in an upregulation of Klotho levels and fast reduction in aortic tissue calcium content with improvement of hemodynamic function.[40]

Although our study was conducted in a carefully phenotyped cohort of ESRD patients, several limitations must be discussed. Firstly, analyses were cross-sectional whereby limiting the possibility of causal inference. Secondly, whereas LD-Rtx recipients are younger, less sedentary and with lower comorbidity, CAC scores and arterial stiffness were on average less severe than in older and sicker prevalent dialysis patients. This reduces the power of analysis by a lower number of cases. Nevertheless, associations between fT3 and CAC scores were of convincing strength. Thirdly, in our analyses we assumed that blood levels of calcification inhibitors and thyroid hormones reflect the situation at a tissue level. This may however be more complex as several modulating steps take place on a tissue level.

In summary, we found fT3, as an indicator of nonthyroidal illness, to be inversely associated with CAC scores and measures of with arterial stiffness in this selected cohort of younger ESRD patients. Whereas fT3 associated positively with serum sKlotho and dp-ucMGP levels, sKlotho, t-ucMGP, dp-ucMGP and PIVKA-II levels did not associate with CAC scores and arterial stiffness. Given the complexity of this association, studies measuring vascular calcification inhibitory status at a tissue level as well as longitudinal studies are needed to provide further clarification on this possible pathway.

## Supporting Information

S1 Appendix Regression analyses on the associations between fT4 and TSH and measures of arterial stiffness/coronary calcification and calcification inhibitors. (DOCX)

S2 Appendix Kärl-Tx data file. (XLSX)
