# Supplementary material for: Associations between Thyroid Hormones, Calcification Inhibitor Levels and Vascular Calcification in End-Stage Renal Disease
Source: PLoS One. 2015 Jul 6;10(7):e0132353. doi: 10.1371/journal.pone.0132353 (PMC4492991; doi:10.1371/journal.pone.0132353)
Supplement: S1 Appendix — (DOCX) [file pone.0132353.s001.docx]

|  | **Log(TSH)** | | | | | | | **fT4** | | | | | |
| --- | --- | --- | --- | --- | --- | --- | --- | --- | --- | --- | --- | --- | --- |
|  | **Crude model** | | | | **Adjusted^1^** | | | **Crude model** | |  | **Adjusted model^1^** | |  |
|  | **Beta (95%CI)** | | **p-value** | | **Beta (95%CI)** | | **p-value** | **Beta (95%CI)** | | **p-value** | **Beta (95%CI)** | | **p-value** |
| **Linear regression analyses** |  | | | |  | |  |  | |  |  | |  |
| **Arterial stiffness** |  | | | |  | |  |  | |  |  | |  |
| **Systolic BP ^#^** | 0.6 | (-3.8 to 5.0) | | 0.785 | 0.2 | (-4.3 to 4.7) | 0.931 | -1.2 | (-2.5 to 0.2) | 0.054 | -1.2 | (-2.5 to 0.0) | 0.054 |
| **Diastolic BP** | 0.1 | (-2.6 to 2.7) | | 0.955 | 0.1 | (-2.4 to 2.7) | 0.927 | -0.9 | (-1.7 to -0.2) | 0.016 | -0.5 | (-1.3 to 0.2) | 0.145 |
| **Pulse pressure** | 0.5 | (-2.7 to 3.7) | | 0.741 | 0.1 | (-3.1 to 3.3) | 0.961 | -0.5 | (-1.4 to 0.5) | 0.313 | -0.7 | (-1.6 to 0.2) | 0.128 |
| **Aortic AP** | -0.1 | (-1.6 to 1.4) | | 0.904 | -0.3 | (-1.8 to 1.2) | 0.699 | 0.2 | (-0.2 to 0.7) | 0.275 | 0.0 | (-0.5 to 0.4) | 0.935 |
| **Aortic Aix** | 0.1 | (-2.5 to 2.7) | | 0.944 | -0.9 | (-3.4 to 1.7) | 0.498 | 0.5 | (-0.3 to 1.2) | 0.197 | 0.1 | (-0.6 to 0.9) | 0.688 |
| **SEVR** | -4.9 | (-13.0 to 3.1) | | 0.227 | -5.9 | (-14.7 to 3.0) | 0.190 | 1.0 | (-1.4 to 3.4) | 0.416 | 0.5 | (-2.1 to 3.1) | 0.684 |
| **Laboratory parameters** |  |  | |  |  | |  |  | |  |  |  |  |
| **Log(t-uc-MGP)** | 0.0 | (-0.1 to 0.1) | | 0.969 | 0.0 | (-0.1 to 0.2) | 0.738 | 0.00 | (-0.03 to 0.04) | 0.854 | 0.01 | (-0.03 to 0.05) | 0.532 |
| **Log(dp-uc-MGP)** | 0.1 | (0.0 to 0.3) | | 0.012 | 0.1 | (0.0 to 0.2) | 0.053 | 0.03 | (-0.03 to 0.03) | 0.795 | 0.00 | (-0.04 to 0.03) | 0.795 |
| **Log(PIVKA)** | 0.0 | (-0.1 to 0.1) | | 0.945 | 0.0 | (-0.2 to 0.2) | 0.924 | 0.00 | (-0.04 to 0.05) | 0.928 | -0.01 | (-0.06 to 0.04) | 0.634 |
| **Log(sKlotho)** | 0.1 | (0.0 to 0.2) | | 0.103 | 0.1 | (0.0 to 0.2) | 0.186 | 0.00 | (-0.03 to 0.04) | 0.785 | 0.01 | (-0.02 to 0.05) | 0.362 |
| **Logistic regression analyses^2^** |  |  | |  |  | |  |  | |  |  | |  |
| **CAC > 100** | 0.6 | (0.3 to 1.3) | | 0.195 | 0.3 | (0.1 to 0.8) | 0.019 | 1.2 | (1.0 to 1.6) | 0.013 | 1.3 | (1.0 to 1.6) | 0.021 |
| **CAC > 400** | 0.6 | (0.3 to 1.3) | | 0.201 | 0.4 | 0.1 to 1.0) | 0.049 | 1.2 | (1.0 to 1.4) | 0.088 | 1.1 | (0.9 to 1.3) | 0.201 |

**S1 Appendix 1. Regression analyses on the associations between fT4 and TSH and measures of arterial stiffness/coronary calcification and calcification inhibitors**

^1^Adjusted for sex, age, diabetes mellitus, IL-6, Vintage, and SGA.

^2^The logistic regression analyses for the association between log(TSH) and fT4 levels and CAC scores were adjusted for age and sex.
